# Targeting Platelet Activation in Abdominal Aortic Aneurysm: Current Knowledge and Perspectives

**DOI:** 10.3390/biom12020206

**Published:** 2022-01-25

**Authors:** Weiliang Sun, Jingang Zheng, Yanxiang Gao

**Affiliations:** 1 Institute of Clinical Medicine Sciences, China-Japan Friendship Hospital, 2 Yinghua Dongjie, Chaoyang District, Beijing 100029, China; sunweiliang@zryhyy.com.cn; 2 Department of Cardiology, China-Japan Friendship Hospital, 2 Yinghua Dongjie, Chaoyang District, Beijing 100029, China; zhengjingang@zryhyy.com.cn

**Keywords:** abdominal aortic aneurysm, platelets, receptors, mediators, intra-luminal thrombus, inflammation, disturbed flow

## Abstract

Abdominal aortic aneurysm (AAA) is a potentially fatal vascular disease that involves complex multifactorial hemodynamic, thrombotic, inflammatory, and aortic wall remodeling processes. However, its mechanisms are incompletely understood. It has become increasingly clear that platelets are involved in pathological processes of vascular diseases beyond their role in hemostasis and thrombosis. Platelet activation with membrane receptors and secreted mediators promotes thrombus formation and the accumulation of inflammatory cells, which may play an important role in the development of AAA by destroying the structural integrity and stability of the vessel wall. Turbulent blood flow in aortic aneurysms promotes platelet activation and aggregation. Platelet count and heterogeneity are important predictive, diagnostic, and prognostic indicators of AAA. We summarize the relationship between platelet activation and AAA development and propose future research directions and possible clinical applications.

## 1. Introduction

Abdominal aortic aneurysm (AAA) is defined as permanent dilatation of the abdominal aorta, which most commonly occurs in the infrarenal region in humans. Although it can have an asymptomatic occurrence, progressive dilation is associated with aortic dissection and rupture [1]. A population ultrasound screening study reported that the prevalence of AAA is 4–8% in males and 0.5–1.5% in females over the age of 65 [2]. The current clinical management of AAA focuses on identifying aneurysms while they are asymptomatic and treating them by endovascular aortic aneurysm repair (EVAR) or open surgery. There are no proven pharmaceutical treatments to prevent progressive growth or rupture. Despite improvements in screening and surgical management, the mortality rates of AAA remain high [3]. A better understanding of AAA development and the emergence of complications is necessary to discover new therapeutic targets.

Abdominal aortic aneurysm is characterized by excessive smooth muscle cell loss, extracellular matrix degradation, and inflammation [4]. Neutrophils, macrophages, and lymphocytes are the main inflammatory cells in AAA, which secrete collagenase, elastase, and cytokines to promote extracellular matrix degradation and smooth muscle cell apoptosis. The increase in proteolytic activity leads to irreversible remodeling of the aortic wall, resulting in aortic expansion and rupture [1,5]. AAA represents a form of atherothrombotic disease, characterized by the formation of a nonocclusive intra-luminal thrombus (ILT) that does not resolve once it occurs. The thromboinflammatory status of ILT contributes to the outward remodeling and eventual disruption of aortic wall integrity [6,7].

The major function of platelets is to contribute to hemostasis and thrombosis [8]. In recent years, many studies have shown that platelets play an important role in the development of AAA [9]. Low platelet count in patients with AAA suggested an increase in platelet consumption, and ILT formation in aortic aneurysm indicated platelet activation [10,11]. Platelet activation participates in AAA pathogenesis via membrane receptors and secreted mediators [12]. Thrombus formation and the accumulation of inflammatory cells and cytokines in ILT may destroy the structural integrity and stability of the vessel wall, thereby increasing the risk for dissection and rupture [6]. Disturbances in flow within the aneurysm sac also further promotes platelet activation and aggregation [13].

In the present review, we focus on basic research and clinical trials that are relevant to the role of platelet activation and its receptors and mediators in the formation and development of AAA, the effects of AAA on platelet activation, and clinical applications that are related to platelets in AAA. Understanding the role of platelets has enabled the continual development of diagnostic biomarkers and possible treatments to optimize clinical outcomes in patients with AAA.

## 2. Methods

We used the PubMed and Science Direct databases to search for scientific information. The terms “platelet”, “platelets”, and “PLT” were combined with the term “abdominal aortic aneurysm” and “AAA” by the Boolean operator “AND”. All titles and abstracts were first screened by the authors to identify potentially relevant studies. We then evaluated the full texts in detail, all original articles, systematic reviews, and meta-analyses identifying relevant abdominal aortic aneurysm in platelet activation were accepted. The references of relevant articles were manually screened to identify additional studies. Studies were excluded if they were not in English, if they were editorials or commentaries, and if they focused on coagulation and the complement system other than platelet activation.

## 3. Results

### 3.1. Study Selection

The PubMed search yielded 411 studies from 1969 to 2021, and the Science Direct search yielded 409 studies that include platelet in the titles and abstracts from 1998 to 2021. After preliminary evaluation of the titles and abstracts, 537 studies were excluded because of the adequacy of its content with the subject matter of the study, 283 studies were further evaluated through their full texts. Finally, we included 106 studies on platelets that were relevant to AAA. We present current knowledge of platelet and vascular hemostasis, the role of platelets, platelet receptors, and platelet-derived mediators in AAA, aortic aneurysm-activated platelets, and the application of platelets in the medical management of AAA.

### 3.2. Platelets and Vascular Hemostasis

Platelets are a component of blood whose function is to react to bleeding from blood vessel injury by clumping. Platelets play an important role in the pathophysiology of thrombosis [14]. Under physiological conditions, thrombus formation on intact endothelial cells is prevented by nitric oxide, adenosine diphosphatase, and prostacyclin. When the endothelial layer is disrupted, collagen and von Willebrand factor (vWF) anchor platelets to the subendothelium. Platelet glycoprotein (GP)Ib/IX/V receptors bind vWF, and GPVI receptors and integrin α2β1 bind collagen. Collagen-mediated GPVI signaling increases the platelet production of thromboxane A2 (TxA2) and decreases the production of prostacyclin [15]. Activated platelets secrete the contents of granules through their canalicular systems to the exterior, including vWF, platelet factor 4 (PF4), platelet-derived growth factor (PDGF), fibrinogen, coagulation factor V, and β-thromboglobulin from alpha granules, calcium, adenine nucleotides, serotonin, pyrophosphate, and polyphosphate from dense granules, and proteases and glycosidases from lysosomal granules [16,17]. Adenosine diphosphate (ADP), vWF, and TxA2 that are released from platelets further promote platelet activation and aggregation [18]. Clot formation occurs as a result of activating GPIIb/IIIa receptors by changing shape to bind fibrinogen. In addition to this classic mechanism, high-velocity blood flow can also initiate aggregation [19]. Clinically, platelet activation can be determined by measuring plasma levels of β-thromboglobulin and PF4 [20].

### 3.3. Role of Platelets in AAA

#### 3.3.1. Low Platelet Count in AAA

Aortic aneurysm is associated with consumption coagulopathy [10]. Cases of complicated AAA with chronic disseminated intravascular coagulopathy have been reported [21,22]. Chronic disseminated intravascular coagulopathy was cured by surgical repair in an elderly patient with AAA over the next 14 days [23]. Although no significant differences were found between acutely symptomatic non-ruptured and ruptured AAA [24], platelet count was significantly lower in patients with AAA compared with healthy controls [25], suggesting an increase in platelet destruction, most likely through activation within the aneurysm sac [26,27]. Patients with AAA had higher baseline spontaneous platelet aggregation compared with normal controls [28]. The relationship between platelet count and aortic aneurysm size is controversial. A clinical study reported that platelet count decreased as aneurysm size increased, and platelet count was lower in patients with a large AAA (diameter > 55 mm) [29]. In contrast, no significant differences in platelet count were found between patients with a large AAA and small AAA in another study [30]. The correlation between platelet indices, such as platelet count, mean platelet volume, the platelet/large cell ratio, and platelet distribution width, are important factors for understanding platelet heterogeneity [31]. In addition to the decrease in platelet count, mean platelet volume, the mean platelet volume-to-platelet count ratio, the mean platelet volume-to-lymphocyte ratio, and the red cell distribution width-to-platelet count ratio were significantly higher in patients with AAA [32].

#### 3.3.2. Platelet-Aggregating Thrombus in AAA

Pathophysiological evidence from patients and animal models indicates ILT formation in the lumen in AAA [33]. The ILT is often structured in three layers in AAA patients: luminal, medial, and abluminal. The luminal ILT layer, which is in contact with blood, is biologically active and enriched in platelets, neutrophils, and red blood cells. The ILT rarely embolizes but does not resolve once it occurs. Eccentric distribution of the ILT was associated with continuous AAA expansion, and a thicker ILT volume was associated with a higher growth rate [34]. Inflammatory cells and cytokines were reported to accumulate in the ILT and play an important role in AAA progression [35,36]. The evolution of ILT can lead to vessel wall weakness through high concentrations of reactive oxygen species, proteases, and cytokines. A study showed that ILT thickness correlated with matrix metalloproteinase 9 (MMP9) expression [37]. Roxana et al. reported that local C3 retention, consumption, and proteolysis in the ILT could induce polymorphonuclear leukocyte chemotaxis and activation, associated with a decrease in systemic complement concentration and activity in later stages of AAA [38].

### 3.4. Platelet Receptors in AAA

**Figure 1:**
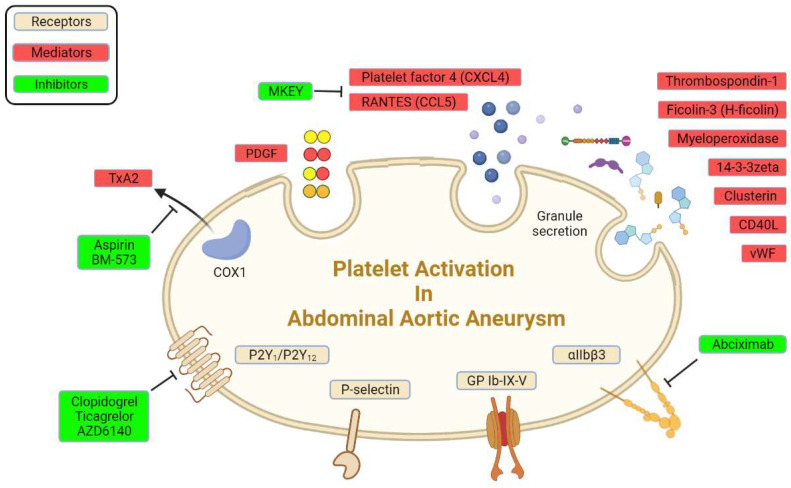
Platelet receptors and derived mediators in AAA.

There are abundant receptors on the surface of platelets that can bind to the extracellular matrix and adhesion proteins to cause platelet adhesion and activation [8]. The platelet membrane has several types of receptors, including integrins (αIIbβ3, α2β5, α5β1, and α2β1), leucine-rich receptors (glycoprotein Ib/IX/V and Toll-like receptors), G-protein-coupled receptors (PAR-1, PAR-4, P2Y12, P2Y1, and TxA2), and C-type lectin receptors (P-selectin), among others [17,39]. Some of these receptors were reported to interact with various extracellular matrices and cells to accelerate AAA progression (Figure 1; Table 1).

#### 3.4.1. Adenosine Diphosphate Receptors

Adenosine diphosphate is an important activator of platelets [40]. It exerts its activity through three major purinergic receptors: P2Y12, P2Y1, and P2X1. They are important for changes in platelet shape and aggregation, TxA2 generation, procoagulant activity, adhesion to immobilized fibrinogen, and thrombus formation under shear conditions. P2Y12 receptors are also important for the potentiation of platelet activation that is mediated by other physiological agonists, including collagen, vWF, and TxA2, resulting in sustained platelet activation [39,41]. P2Y12 receptor antagonists, such as clopidogrel and ticagrelor, inhibit platelets by selectively and irreversibly binding to P2Y12 receptors and blocking the ADP-dependent pathway of platelet activation. In a rat model that implanted a segment of the sodium dodecyl sulfate-decellularized guinea pig aorta, the P2Y12 receptor antagonist AZD6140 inhibited platelet activation and prevented the development of AAA by inhibiting ADP-induced platelet aggregation and limiting biological activity of the ILT [42]. Administration of the P2Y12 inhibitor clopidogrel significantly suppressed aortic expansion, elastic lamina degradation, inflammatory cytokine expression, and aortic aneurysm rupture in an established animal model of AAA that was induced by Ang II infusion in hypercholesterolemic mice [43]. Clopidogrel bisulfate reduced death among patients with AAA [44]. However, a multicenter randomized, double-blind, controlled trial of ticagrelor and placebo reported different results. Patients who were randomized to ticagrelor did not exhibit a reduction in AAA growth compared with controls during the 12-month follow-up period. This was the first interventional trial on AAA growth using AAA volume as the primary outcome measure other than rupture [45]. In response to the limited clinical studies of the effects of ADP receptor antagonists on AAA, a Phase 2 clinical trial is currently verifying the efficacy of ticagrelor in patients with a small AAA (ClinicalTrials identifier: NCT02070653).

#### 3.4.2. P-Selectin

P-selectin (CD62P) is an adhesion receptor for neutrophils and macrophages that is expressed on both endothelial cells and platelets [46]. In platelets, P-selectin is stored in α granules and mobilized to the external plasma membrane within minutes after activation. The expression of P-selectin on activated platelets is important for the recruitment of leukocytes to thrombi and induction of fibrin production during hemostasis. Because detection is relatively easy, soluble P-selectin was assayed as a marker of platelet activity. Soluble P-selectin significantly increased in plasma in patients with AAA [36]. Likewise, P-selectin significantly increased in an animal model of AAA that was established by xenografting a segment of the sodium dodecyl sulfate-decellularized guinea-pig aorta (xenogenic matrix) onto the abdominal aorta in rats [36]. P-selectin deficiency attenuated AAA formation in elastase aortic perfusion mice, with diminished aortic wall degradation and preserved elastin and collagen [47]. P-selectin glycoprotein ligand-1 (PSGL-1) acts as a critical regulator of inflammatory cells infiltration by mediating the adhesion of leukocytes. PSGL-1 deficiency reduced the incidence and severity of AAA by inhibiting inflammatory cell migration and recruitment under conditions of aortic aneurysm [48].

#### 3.4.3. Other Receptors

Integrin αIIbβ3 is expressed at high levels in platelets and their progenitors. In resting platelets, integrin αIIbβ3 adopts an inactive conformation. Upon agonist stimulation, it switches from a low- to high-affinity state for fibrinogen and other ligands. Ligand binding causes integrin clustering and subsequently promotes outside-in signaling, which drives essential platelet functions, such as spreading, aggregation, clot retraction, and thrombus consolidation [49]. The inhibition of integrin αIIbβ3 by treatment with the Fab fragment abciximab for 6 weeks reduced both thrombus area and aneurysmal enlargement in a rat xenograft model of AAA compared with treatment with irrelevant immunoglobulins [36].

GPIb is a major glycoprotein on the platelet surface. Like the integrin αIIbβ3, GPIb undergoes reversible translocation as a function of platelet activation [50]. As an extramembranous portion of GPIb, glycocalicin was higher in patients with AAA than in patients who underwent carotid endarterectomy, indicating that GPIb was cleaved from the platelet membrane after platelet activation and turnover [26].

**Table 1:** Characteristics of studies of platelet receptors in AAA.

Target	Inhibitor	Disease Model	Study Type	Main Findings	Reference
ADP receptor	P2Y12 receptor antagonist AZD6140	Decellularized aortic xenograft model of AAA in rats	Animal study	Reduced the spontaneous increase in aortic diameter	[42]
ADP receptor	Clopidogrel	Apolipoprotein E-knockout mice infused with Ang II (AAA model)	Animal study	Suppressed aneurysm formation	[43]
ADP receptor	Clopidogrel bisulfate	Hypercholesterolemic mice infused with Ang II (AAA model)	Animal study	Reduced AAA rupture	[44]
ADP receptor	Clopidogrel bisulfate, ticagrelor, or prasugrel	Patients with AAA who progressed to rupture or dissection	Cohort study	Reduced rupture and dissection	[44]
ADP receptor	Ticagrelor	Patients with AAA and a maximum aorta diameter of 35–49 mm	Multicenter randomized controlled trial	No reduction in growth of small AAA	[45]
P-selectin	—	Patients with AAA before surgery	Cohort study	Soluble P-selectin significantly increased in plasma	[36]
P-selectin	—	Decellularized aortic xenograft model of AAA in rats	Animal study	Soluble P-selectin significantly increased in rats	[36]
P-selectin	Global knockout	P-selectin knockout mice with elastase perfusion (AAA model)	Animal study	P-selectin deficiency attenuated aneurysm formation	[47]
P-selectin	Global PSGL-1 knockout	Aortic aneurysm model induced by deoxycorticosterone acetate plus high salt	Animal study	Reduced the incidence and severity of aortic aneurysm	[48]
αIIbβ3	αIIbβ3 inhibitor abciximab	Decellularized aortic xenograft model of AAA in rats	Animal study	Reduced thrombus area and aneurysmal enlargement	[36]
GPIb	—	Patients with asymptomatic AAA	Case-control study	Higher glycocalicin produced by cleaved GPIb than normal population	[26]

### 3.5. Platelet-Derived Mediators in AAA

Extensive research has revealed several mediators that are released from activated platelets. Activated platelets release prothrombotic mediators from dense granules and alpha granules, including vWF and ADP, and newly synthesized TxA2 [18]. Microparticles that derived from platelets significantly increased in AAA eluates in rats and plasma in patients [36]. These mediators orchestrate the development and progression of AAA (Figure 1) by enhancing the interaction between platelets and extracellular matrix and other cells (Table 2).

#### 3.5.1. TxA2

TxA2 is produced from arachidonate through the aspirin-sensitive cyclooxygenase 1 (COX-1) pathway in activated platelets and involved in multiple biological processes via its cell-surface thromboxane prostanoid (TP) receptor. TxA2 binds to TP, leading to changes in platelet shape, phospholipase A2 activation, the platelet degranulation of dense granules and alpha granules, and platelet aggregation [51]. The release of TxA2 amplifies the initial stimulus for platelet activation and helps recruit additional platelets. TxB2 (the hydrolysis product of TxA2) was the main prostanoid that was produced by tissue from AAA in humans [52]. Preliminary promising results indicated that the TxA2 inhibitor BM-573 suppressed aneurysmal growth in rats [53].

Aspirin (acetylsalicylic acid) irreversibly inhibits platelet COX-1, blocking TxA2 production in platelets and decreasing platelet aggregation. The administration of aspirin dramatically reduced the rupture of AAA that was induced by a high-fat diet and Ang II infusion in Ldlr^−/−^ mice. Aspirin also reduced platelet and macrophage recruitment, resulting in a decrease in MMP activity, and reduced plasma concentrations of PF4, cytokines, and components of the plasminogen activation system in abdominal aortas in mice [44]. Patients with AAA are recommended to receive low-dose aspirin or a P2Y12 receptor antagonist for the secondary prevention of AAA progression or rupture [54,55].

#### 3.5.2. PDGF

PDGF is a potent mitogen for cells that is partially synthesized and stored in alpha granules of platelets and released upon platelet activation. There are five different isoforms of PDGF that activate cellular responses through two different receptors (PDGFRα and PDGFRβ), known as PDGF-AA (PDGFA), PDGF-BB (PDGFB), PDGF-CC (PDGFC), PDGF-DD (PDGFD), and PDGF-AB [56]. PDGFA was reported to increase in human AAA tissue in a membrane-based complementary DNA expression array [57]. PDGF A and B chains were strongly stained on small vessels in aneurysmal walls of atherosclerotic AAA in patients, whereas the weaker expression of PDGF A and B chains was observed in endothelial cells of vessel walls around inflammatory cells in the aneurysmal wall of inflammatory AAA [58]. PDGFD was shown to mediate adventitial inflammation, which provided a direct link between perivascular adipose tissue dysfunction and AAA formation in Ang II-infused obese mice [59].

#### 3.5.3. CD40L-CD40

Both CD40L and CD40 exist in platelets [60]. CD40L is cryptic in unstimulated platelets but rapidly presents to the platelet surface after platelet activation. CD40L that is expressed on the cell surface is subsequently cleaved, generating a soluble fragment, termed sCD40L. Studies of the cellular distribution of CD40L indicated that >95% of circulating CD40L was produced by platelets [61,62]. The ligation of platelet CD40 with sCD40L increased platelet P-selectin expression and granule release and enhanced platelet-leukocyte adhesion [63]. The abundance of both CD40L and CD40 increased in media of thrombus-free and thrombus-covered human AAA samples. The CD40L–CD40 axis has been implicated in aneurysm formation. CD40L deficiency reduced inflammatory chemokine/cytokine expression, MMP activity, and macrophage infiltration, lowering the incidence of AAA and risk of rupture [64].

#### 3.5.4. Platelet Factor 4 (PF4/CXCL4) and RANTES (CCL5)

Platelets regulate leukocyte recruitment indirectly via the release of chemokines upon platelet activation. Platelet factor 4 (PF4/CXCL4) and RANTES (CCL5) are two main chemokines that are located within alpha granules of platelets. CXCL4 has been shown to form functional heterodimers with CCL5 to promote the recruitment of neutrophils, macrophages, and T cells [65]. CXCL4 and CCL5 levels increased in plasma in AAA patients, with high levels in luminal layers of ILTs [66]. Platelets and neutrophils co-localized in luminal ILT layers of AAA [66], and plasma levels of CXCL4 and CCL5 were positively associated with macrophage recruitment in murine AAA models [44]. The inhibition of CXCL4-CCL5 heterodimers by the peptide inhibitor MKEY before or after the induction of experimental AAA was reported to efficiently prevent the development of AAA or halt its progression, respectively [67].

#### 3.5.5. Other Mediators

Ficolin-3 (H-ficolin) is one of the most abundant and efficient recognition molecules in the lectin pathway of the complement system, which was identified in platelet-derived microvesicles [68]. Ficolin-3 levels in microvesicles that were obtained from plasma-activated platelets and AAA tissue were associated with the presence and progression of AAA compared with healthy ones [69]. High levels of ficolin-3 in the AAA thrombus could be involved in complement-coagulation crosstalk and the immune-inflammatory response that is associated with AAA.

Myeloperoxidase, which is partially released by activated platelets, was localized both on the surface of and inside platelets. Etienne et al. found that myeloperoxidase was significantly elevated in experimental saccular aneurysms compared with fusiform aneurysms in a decellularized xenograft model in rats [70], and it caused oxidative damage by producing superoxide in a chronic remodel of AAA [71].

vWF is a large, multimeric glycoprotein that is found in blood plasma, platelet granules, and subendothelial connective tissue that mediates the adhesion of platelets to subendothelial connective tissue [72]. A prospective study found that vWF activity in plasma correlated with AAA thrombus size [73]. Another study found that 14-3-3ζ that was stored in dense granules was secreted by activated platelets in the abdominal aorta in patients with aneurysm, based on an organellar proteomics method [74]. Serum levels of thrombospondin-1 and clusterin, which are secreted by platelets, were negatively associated among 1003 AAA patients [75].

**Table 2:** Characteristics of studies of platelet-derived mediators in AAA.

Target	Inhibitor	Disease Model	Study Type	Main Findings	Reference
TxA2	TxA2 inhibitor BM-573	AAA model in rats	Animal study	Suppressed aneurysmal growth	[53]
TxA2	Aspirin	Ang II infusion in hypercholesterolemic mice (AAA model)	Animal study	Reduced rupture	[44]
PDGFA	—	Patients with AAA	Cohort study	Increased in AAA tissue	[57]
PDGFA, PDGFB	—	Patients with AAA	Cohort study	Stained on small vessels in aneurysmal walls	[58]
PDGFD	—	Ang II-infused obese mice (AAA model)	Animal study	Inhibition in PDGFD function significantly reduced the incidence of AAA	[59]
CD40L	CD40L global knockout	Ang II infusion (AAA model)	Animal study	Developed fewer aneurysms	[64]
PF4 and RANTES	—	Patients with AAA	Cohort study	Involved in attracting neutrophils to the luminal layer of AAA specimens	[66]
PF4 and RANTES	MKEY, peptide inhibitor of CXCL4-CCL5	Transient infrarenal aortic porcine pancreatic elastase infusion in mice (AAA model)	Animal study	Reduced aortic diameter enlargement	[67]
Ficolin-3	—	Patients with AAA	Cohort study	Increased from activated platelets and AAA tissue	[69]
PF4 and myeloperoxidase	—	Decellularized aortic xenograft model in rats (AAA model)	Animal study	Elevated in experimental saccular aneurysm compared with fusiform aneurysm	[70,71]
vWF	—	Patients with AAA	Cohort study	Elevated pre- and postoperatively, decreased intraoperatively	[73]
14-3-3ζ	—	Patients with AAA	Cohort study	Elevated in sections of AAA specimens	[74]
Thrombospondin-1 and clusterin	—	Patients with AAA	Cohort study	Negatively associated with AAA patients in serum	[75]

### 3.6. Platelet Activation and Hemodynamic Changes in AAA

From an engineering perspective, the generation of aortic aneurysm is a failure of the aorta to withstand hemodynamic forces [76]. Using patient-specific geometries that were derived from computed tomography, computational fluid dynamics has emerged as a powerful and popular tool for studying blood flow dynamics of AAA [77,78]. By modeling platelets as infinitesimal and finite-sized particles or even as a continuum quantity, the biomechanical and biochemical activation potential of tracked platelets was quantified.

Much attention has focused on studying hemodynamics in AAA. Using a numerical simulation of flow through an axisymmetric aneurysm under laminar and turbulent steady flow conditions, the recirculation zone formed inside the aneurysm cavity creates conditions that promote platelet deposition and thrombus formation in vitro [79]. A novel computational particle-hemodynamics analysis of platelet residence times showed high potential to entrap activated blood particles in a patient-specific AAA [80]. In contrast to the normal aorta, the flow in an aneurysm was highly disturbed. Flow separation that involved regions of high streaming velocities and high shear stress was observed where platelets exhibited adhesion and activation [19]. Biasetti et al. reported a fluid-dynamics-motivated mechanism of platelet activation, convection, and deposition in AAAs [81]. A reliable three-dimensional flow visualization method indicated that a longer residence time of recirculated blood flow in the aortic lumen that is caused by this vortex caused sufficient shear-induced platelet activation to develop ILT and maintain uniform flow conditions [82]. Patient-specific computational fluid dynamic models were used to analyze stress-induced platelet activation within AAA under physiological conditions [83].

### 3.7. Clinical Applications Related to Platelets in AAA

#### 3.7.1. Labeled Platelets and Visualization Methods

Labeled platelets and visualization method reveal the role of the platelet activation in aneurysm progression in another way. Accompanied by platelet activation, phosphatidylserine that is exposed on platelet membranes is a mediator that links platelet vesicles to aneurysm progression. Radiolabeled 99mTc-annexin-V specifically binds phosphatidylserine and has been used to assess the renewal activity of ILT in an in vivo experimental model of AAA and ex vivo in human ILT [84]. 99mTc-fucoidan is an imaging agent for the in vivo detection of biological activity that is associated with P-selectin overexpression on activated platelets in humans and rats with AAA [85,86]. Biodegradable microcapsules that are made of polycyanoacrylate and polysaccharide that are functionalized with fucoidan had high binding activities by targeting arterial thrombi that overexpressed P-selectin in human activated platelets and rat AAA thrombotic wall [87].

#### 3.7.2. Platelets and Surgical Interventions

Surgical repair, including traditional open surgical repair and EVAR, is indicated for AAA with a diameter greater than 5.5 cm in men and 5.0 cm in women, growth of more than 0.5 cm in 6 months, or AAA-related symptoms, such as rupture, dissection, and pain [88]. Low platelet count at the time of hospital admission predicts poor outcome in patients who undergo the emergency repair of a ruptured AAA [89,90]. Platelet count and platelet activity significantly increased after AAA repair [25,91]. Platelet count decreased significantly in patients who underwent EVAR during the first few days postsurgery, returning to preoperative levels by 1-week to 1-month post-EVAR [92,93,94]. Vascular surgeons encounter an endovascular-specific problem, the so-called endoleak, which reduces the curability of EVAR. In EVAR in 249 patients, platelet count after EVAR in patients with malignant type II endoleak was lower than in patients without malignant endoleak [95]. A lack of aneurysm shrinkage by 7 days and 6 months after EVAR was significantly associated with ongoing multiagent antiplatelet therapy with clopidogrel, ticlopidine, cilostazol, and aspirin [96].

#### 3.7.3. Platelet Infusion and Perioperative Period

Transfusion during open surgery is essential to increase platelet count and function in response to massive blood loss and platelet disorders. Patients with ruptured AAA who received proactive transfusion therapy with platelets had a higher platelet count when they were admitted to the intensive care unit compared with the control group [97]. Patients with ruptured AAA who received more platelets and plasma intraoperatively had lower 30-day mortality compared with control patients [98]. Patients who were scheduled to undergo the open repair of a ruptured AAA, however, received no significant benefit from the early administration of platelets with regard to postoperative complications and mortality [99]. Platelet transfusion was an independent marker of thrombotic complications in patients with ruptured AAA [100].

## 4. Concluding Remarks and Future Perspectives

Accumulating data from animal studies and clinical observations demonstrate that platelets contribute to the formation, progression, and rupture of AAA, in addition to their physiological functions in stopping bleeding and maintaining vascular integrity. Receptors and mediators that are released from activated platelets mediate interactions between platelets and the inflammatory cells/matrix. Disturbances in blood flow in aortic aneurysm activate platelets and promote ILT formation. The inhibition of platelet activation, such as by reducing TxA2 secretion and applying P2Y12 inhibitors, may restrain the development of AAA. These positive preclinical findings remain to be confirmed in clinical trials.

Several challenges restrict further progress in platelet research in AAA. First, AAA is believed to result from a combination of inherited and environmental factors that trigger a complex thrombotic and inflammatory disorder, leading to a wide diversity of mechanisms [101]. Second, the acquisition of tissue in excess of 5 cm that is grossly distorted restricts clear descriptive pathologies in clinical practice. Animal models that mimic cellular and biochemical characteristics of human disease progression are needed. In cases of a lack of spontaneous ILT formation in the majority of aneurysm models [102,103,104], only aortic elastase perfusion and xenograft models produce ILT that is similar to saccular aneurysm [105]. This diversity allows one to focus only on each specific mechanism that is involved in AAA development. Third, global knockout mouse models are used in many experimental studies, meaning that existing data are not platelet specific. Further research could be restricted to platelet-free plasma because platelets release many substances that remain in serum during coagulation [106]. Fourth, circulating chemokine levels are produced by various cell types beyond platelets. Further attention should be given to platelet-specific targets in experimental and clinical studies that potentially contribute to assessments of prognosis. Further studies should also be conducted with newer classes of anti-platelet therapies.

Current guidelines suggest that antiplatelet drugs should be prescribed in all patients with AAA to reduce the cardiovascular risk of morbidity and mortality. Data show, however, that antiplatelet drugs may have no effect or even increase the risk of bleeding. The evidence still seems to be contradictory and has insufficient validity. Randomized controlled trials with longer follow-up times should be conducted to assess the efficacy of antiplatelet medications in reducing aneurysm progression in non-surgery intervention patients.

## Data Availability

The study did not report any data.

## Abbreviations

AAA	abdominal aortic aneurysm
ADP	adenosine diphosphate
Ang II	angiotensin II
COX-1	cyclooxygenase 1
EVAR	endovascular aortic aneurysm repair
GP	glycoprotein
ILT	intra-luminal thrombus
MMP	matrix metalloproteinase
PDGF	platelet-derived growth factor
PF4	platelet factor 4
PSGL-1	P-selectin glycoprotein ligand-1
TP	thromboxane prostanoid
TxA2	thromboxane A2
vWF	von Willebrand factor

## References

[1] Golledge J., Muller J., Daugherty A., Norman P. (2006). Abdominal aortic aneurysm: Pathogenesis and implications for management. Arter. Thromb. Vasc. Biol..

[2] Eckstein H.H., Bockler D., Flessenkamper I., Schmitz-Rixen T., Debus S., Lang W. (2009). Ultrasonographic screening for the detection of abdominal aortic aneurysms. Dtsch Arztebl Int..

[3] Golledge J., Powell J.T. (2007). Medical management of abdominal aortic aneurysm. Eur. J. Vasc. Endovasc Surg..

[4] Sakalihasan N., Limet R., Defawe O.D. (2005). Abdominal aortic aneurysm. Lancet.

[5] Nordon I.M., Hinchliffe R.J., Loftus I.M., Thompson M.M. (2011). Pathophysiology and epidemiology of abdominal aortic aneurysms. Nat. Rev. Cardiol..

[6] Kazi M., Thyberg J., Religa P., Roy J., Eriksson P., Hedin U., Swedenborg J. (2003). Influence of intraluminal thrombus on structural and cellular composition of abdominal aortic aneurysm wall. J. Vasc. Surg..

[7] Behr-Rasmussen C., Grondal N., Bramsen M.B., Thomsen M.D., Lindholt J.S. (2014). Mural thrombus and the progression of abdominal aortic aneurysms: A large population-based prospective cohort study. Eur. J. Vasc. Endovasc. Surg..

[8] Yip J., Shen Y., Berndt M.C., Andrews R.K. (2005). Primary platelet adhesion receptors. IUBMB Life.

[9] Schrottmaier W.C., Mussbacher M., Salzmann M., Assinger A. (2020). Platelet-leukocyte interplay during vascular disease. Atherosclerosis.

[10] Bieger R., Vreeken J., Stibbe J., Loeliger E.A. (1971). Arterial aneurysm as a cause of consumption coagulopathy. N. Engl. J. Med..

[11] Boyd A.J. (2021). Intraluminal thrombus: Innocent bystander or factor in abdominal aortic aneurysm pathogenesis?. JVS Vasc. Sci..

[12] Li Z., Delaney M.K., O’Brien K.A., Du X. (2010). Signaling during platelet adhesion and activation. Arterioscler. Thromb. Vasc. Biol..

[13] Goto S. (2004). Understanding the mechanism of platelet thrombus formation under blood flow conditions and the effect of new antiplatelet agents. Curr. Vasc. Pharmacol..

[14] Davi G., Patrono C. (2007). Platelet activation and atherothrombosis. N. Engl. J. Med..

[15] Gremmel T., Frelinger A.L., Michelson A.D. (2016). Platelet Physiology. Semin. Thromb. Hemost..

[16] Rubenstein D.A., Yin W. (2018). Platelet-Activation Mechanisms and Vascular Remodeling. Compr. Physiol..

[17] George J.N. (2000). Platelets. Lancet.

[18] Savage B., Shattil S.J., Ruggeri Z.M. (1992). Modulation of platelet function through adhesion receptors. A dual role for glycoprotein IIb-IIIa (integrin alpha IIb beta 3) mediated by fibrinogen and glycoprotein Ib-von Willebrand factor. J. Biol. Chem..

[19] Biasetti J., Gasser T.C., Auer M., Hedin U., Labruto F. (2010). Hemodynamics of the normal aorta compared to fusiform and saccular abdominal aortic aneurysms with emphasis on a potential thrombus formation mechanism. Ann. Biomed. Eng..

[20] Lane D.A., Ireland H., Wolff S., Ranasinghe E., Dawes J. (1984). Detection of enhanced in vivo platelet alpha-granule release in different patient groups--comparison of beta-thromboglobulin, platelet factor 4 and thrombospondin assays. Thromb. Haemost..

[21] Mukaiyama H., Shionoya S., Ikezawa T., Kamiya T., Hamaguchi M., Saito H. (1987). Abdominal aortic aneurysm complicated with chronic disseminated intravascular coagulopathy: A case of surgical treatment. J. Vasc. Surg..

[22] Otsui K., Yamamoto M., Aoki H., Ozawa T., Domoto K., Suzuki A., Iwata S., Takei A., Inamoto S., Inoue N. (2015). A super-elderly case of abdominal aortic aneurysm associated with chronic disseminated intravascular coagulation. J. Cardiol. Cases.

[23] Rowlands T.E., Norfolk D., Homer-Vanniasinkam S. (2000). Chronic disseminated intravascular coagulopathy cured by abdominal aortic aneurysm repair. Cardiovasc. Surg..

[24] Adam D.J., Haggart P.C., Ludlam C.A., Bradbury A.W. (2002). Hemostatic markers before operation in patients with acutely symptomatic nonruptured and ruptured infrarenal abdominal aortic aneurysm. J. Vasc. Surg..

[25] Yamazumi K., Ojiro M., Okumura H., Aikou T. (1998). An activated state of blood coagulation and fibrinolysis in patients with abdominal aortic aneurysm. Am. J. Surg..

[26] Milne A.A., Adam D.J., Murphy W.G., Ruckley C.V. (1999). Effects of asymptomatic abdominal aortic aneurysm on the soluble coagulation system, platelet count and platelet activation. Eur. J. Vasc. Endovasc. Surg..

[27] Singh K., Bonaa K.H., Jacobsen B.K., Bjork L., Solberg S. (2001). Prevalence of and risk factors for abdominal aortic aneurysms in a population-based study: The Tromso Study. Am. J. Epidemiol..

[28] Robless P.A., Okonko D., Lintott P., Mansfield A.O., Mikhailidis D.P., Stansby G.P. (2003). Increased platelet aggregation and activation in peripheral arterial disease. Eur. J. Vasc. Endovasc. Surg..

[29] Flondell-Site D., Lindblad B., Kolbel T., Gottsater A. (2009). Cytokines and systemic biomarkers are related to the size of abdominal aortic aneurysms. Cytokine.

[30] Wallinder J., Bergqvist D., Henriksson A.E. (2009). Haemostatic markers in patients with abdominal aortic aneurysm and the impact of aneurysm size. Thromb. Res..

[31] Ihara A., Matsumoto K., Kawamoto T., Shouno S., Kawamoto J., Katayama A., Yoshitatsu M., Izutani H. (2006). Relationship between hemostatic markers and platelet indices in patients with aortic aneurysm. Pathophysiol. Haemost. Thromb..

[32] Tekin Y.K., Tekin G. (2020). Mean Platelet Volume-to-Platelet Count Ratio, Mean Platelet Volume-to-Lymphocyte Ratio, and Red Blood Cell Distribution Width-Platelet Count Ratio as Markers of Inflammation in Patients with Ascending Thoracic Aortic Aneurysm. Braz. J. Cardiovasc. Surg..

[33] Michel J.B., Martin-Ventura J.L., Egido J., Sakalihasan N., Treska V., Lindholt J., Allaire E., Thorsteinsdottir U., Cockerill G., Swedenborg J. (2011). Novel aspects of the pathogenesis of aneurysms of the abdominal aorta in humans. Cardiovasc. Res..

[34] Speelman L., Schurink G.W., Bosboom E.M., Buth J., Breeuwer M., van de Vosse F.N., Jacobs M.H. (2010). The mechanical role of thrombus on the growth rate of an abdominal aortic aneurysm. J. Vasc. Surg..

[35] Fontaine V., Jacob M.P., Houard X., Rossignol P., Plissonnier D., Angles-Cano E., Michel J.B. (2002). Involvement of the mural thrombus as a site of protease release and activation in human aortic aneurysms. Am. J. Pathol..

[36] Touat Z., Ollivier V., Dai J., Huisse M.G., Bezeaud A., Sebbag U., Palombi T., Rossignol P., Meilhac O., Guillin M.C. (2006). Renewal of mural thrombus releases plasma markers and is involved in aortic abdominal aneurysm evolution. Am. J. Pathol..

[37] Khan J.A., Abdul Rahman M.N., Mazari F.A., Shahin Y., Smith G., Madden L., Fagan M.J., Greenman J., McCollum P.T., Chetter I.C. (2012). Intraluminal thrombus has a selective influence on matrix metalloproteinases and their inhibitors (tissue inhibitors of matrix metalloproteinases) in the wall of abdominal aortic aneurysms. Ann. Vasc. Surg..

[38] Martinez-Pinna R., Madrigal-Matute J., Tarin C., Burillo E., Esteban-Salan M., Pastor-Vargas C., Lindholt J.S., Lopez J.A., Calvo E., de Ceniga M.V. (2013). Proteomic analysis of intraluminal thrombus highlights complement activation in human abdominal aortic aneurysms. Arterioscler. Thromb. Vasc. Biol..

[39] Ibrahim H., Kleiman N.S. (2017). Platelet pathophysiology, pharmacology, and function in coronary artery disease. Coron. Artery Dis..

[40] Wijeyeratne Y.D., Heptinstall S. (2011). Anti-platelet therapy: ADP receptor antagonists. Br. J. Clin. Pharmacol..

[41] Dorsam R.T., Kunapuli S.P. (2004). Central role of the P2Y12 receptor in platelet activation. J. Clin. Investig..

[42] Dai J., Louedec L., Philippe M., Michel J.B., Houard X. (2009). Effect of blocking platelet activation with AZD6140 on development of abdominal aortic aneurysm in a rat aneurysmal model. J. Vasc. Surg..

[43] Liu O., Jia L., Liu X., Wang Y., Wang X., Qin Y., Du J., Zhang H. (2012). Clopidogrel, a platelet P2Y12 receptor inhibitor, reduces vascular inflammation and angiotensin II induced-abdominal aortic aneurysm progression. PLoS ONE.

[44] Owens A.P., Edwards T.L., Antoniak S., Geddings J.E., Jahangir E., Wei W.Q., Denny J.C., Boulaftali Y., Bergmeier W., Daugherty A. (2015). Platelet Inhibitors Reduce Rupture in a Mouse Model. of Established Abdominal Aortic Aneurysm. Arterioscler. Thromb. Vasc. Biol..

[45] Wanhainen A., Mani K., Kullberg J., Svensjo S., Bersztel A., Karlsson L., Holst J., Gottsater A., Linne A., Gillgren P. (2020). The effect of ticagrelor on growth of small abdominal aortic aneurysms-a randomized controlled trial. Cardiovasc. Res..

[46] Andre P. (2004). P-selectin in haemostasis. Br. J. Haematol..

[47] Hannawa K.K., Cho B.S., Sinha I., Roelofs K.J., Myers D.D., Wakefield T.J., Stanley J.C., Henke P.K., Upchurch G.R. (2006). Attenuation of experimental aortic aneurysm formation in P-selectin knockout mice. Ann. N. Y. Acad. Sci..

[48] Wu X., Liu X., Yang H., Chen Q., Zhang N., Li Y., Du X., Liu X., Jiang X., Jiang Y. (2021). P-Selectin Glycoprotein Ligand-1 Deficiency Protects Against Aortic Aneurysm Formation Induced by DOCA Plus Salt. Cardiovasc. Drugs Ther..

[49] Huang J., Li X., Shi X., Zhu M., Wang J., Huang S., Huang X., Wang H., Li L., Deng H. (2019). Platelet integrin alphaIIbbeta3: Signal transduction, regulation, and its therapeutic targeting. J. Hematol. Oncol..

[50] Ozaki Y., Asazuma N., Suzuki-Inoue K., Berndt M.C. (2005). Platelet GPIb-IX-V-dependent signaling. J. Thromb. Haemost..

[51] Fontana P., Zufferey A., Daali Y., Reny J.L. (2014). Antiplatelet therapy: Targeting the TxA2 pathway. J. Cardiovasc. Transl. Res..

[52] Ritter J.M., Frazer C.E., Powell J.T., Taylor G.W. (1988). Prostaglandin and thromboxane synthesis by tissue slices from human aortic aneurysms. Prostaglandins Leukot Essent Fat. Acids.

[53] Courtois A., Makrygiannis G., Cheramy-Bien J.P., Purnelle A., Pirotte B., Dogne J.M., Hanson J., Defraigne J.O., Drion P., Sakalihasan N. (2018). Therapeutic Applications of Prostaglandins and Thromboxane A2 Inhibitors in Abdominal Aortic Aneurysms. Curr. Drug Targets..

[54] Hirsch A.T., Haskal Z.J., Hertzer N.R., Bakal C.W., Creager M.A., Halperin J.L., Hiratzka L.F., Murphy W.R., Olin J.W., Puschett J.B. (2006). ACC/AHA 2005 Practice Guidelines for the management of patients with peripheral arterial disease (lower extremity, renal, mesenteric, and abdominal aortic): A collaborative report from the American Association for Vascular Surgery/Society for Vascular Surgery, Society for Cardiovascular Angiography and Interventions, Society for Vascular Medicine and Biology, Society of Interventional Radiology, and the ACC/AHA Task Force on Practice Guidelines (Writing Committee to Develop Guidelines for the Management of Patients With Peripheral Arterial Disease): Endorsed by the American Association of Cardiovascular and Pulmonary Rehabilitation; National Heart, Lung, and Blood Institute; Society for Vascular Nursing; TransAtlantic Inter.-Society Consensus; and Vascular Disease Foundation. Circulation.

[55] Moll F.L., Powell J.T., Fraedrich G., Verzini F., Haulon S., Waltham M., van Herwaarden J.A., Holt P.J., van Keulen J.W., Rantner B. (2011). Management of abdominal aortic aneurysms clinical practice guidelines of the European society for vascular surgery. Eur. J. Vasc. Endovasc. Surg..

[56] Fredriksson L., Li H., Eriksson U. (2004). The PDGF family: Four gene products form five dimeric isoforms. Cytokine Growth Factor Rev..

[57] Tung W.S., Lee J.K., Thompson R.W. (2001). Simultaneous analysis of 1176 gene products in normal human aorta and abdominal aortic aneurysms using a membrane-based complementary DNA expression array. J. Vasc. Surg..

[58] Kanazawa S., Miyake T., Kakinuma T., Tanemoto K., Tsunoda T., Kikuchi K. (2005). The expression of platelet-derived growth factor and connective tissue growth factor in different types of abdominal aortic aneurysms. J. Cardiovasc. Surg. (Torino).

[59] Zhang Z.B., Ruan C.C., Lin J.R., Xu L., Chen X.H., Du Y.N., Fu M.X., Kong L.R., Zhu D.L., Gao P.J. (2018). Perivascular Adipose Tissue-Derived PDGF-D Contributes to Aortic Aneurysm Formation During Obesity. Diabetes.

[60] Henn V., Steinbach S., Buchner K., Presek P., Kroczek R.A. (2001). The inflammatory action of CD40 ligand (CD154) expressed on activated human platelets is temporally limited by coexpressed CD40. Blood.

[61] Santilli F., Basili S., Ferroni P., Davi G. (2007). CD40/CD40L system and vascular disease. Intern. Emerg. Med..

[62] Andre P., Prasad K.S., Denis C.V., He M., Papalia J.M., Hynes R.O., Phillips D.R., Wagner D.D. (2002). CD40L stabilizes arterial thrombi by a beta3 integrin--dependent mechanism. Nat. Med..

[63] Inwald D.P., McDowall A., Peters M.J., Callard R.E., Klein N.J. (2003). CD40 is constitutively expressed on platelets and provides a novel mechanism for platelet activation. Circ. Res..

[64] Kusters P.J.H., Seijkens T.T.P., Beckers L., Lievens D., Winkels H., de Waard V., Duijvestijn A., Lindquist Liljeqvist M., Roy J., Daugherty A. (2018). CD40L Deficiency Protects Against Aneurysm Formation. Arterioscler. Thromb. Vasc. Biol..

[65] Bakogiannis C., Sachse M., Stamatelopoulos K., Stellos K. (2019). Platelet-derived chemokines in inflammation and atherosclerosis. Cytokine.

[66] Houard X., Touat Z., Ollivier V., Louedec L., Philippe M., Sebbag U., Meilhac O., Rossignol P., Michel J.B. (2009). Mediators of neutrophil recruitment in human abdominal aortic aneurysms. Cardiovasc. Res..

[67] Iida Y., Xu B., Xuan H., Glover K.J., Tanaka H., Hu X., Fujimura N., Wang W., Schultz J.R., Turner C.R. (2013). Peptide inhibitor of CXCL4-CCL5 heterodimer formation, MKEY, inhibits experimental aortic aneurysm initiation and progression. Arterioscler. Thromb. Vasc. Biol..

[68] Garred P., Genster N., Pilely K., Bayarri-Olmos R., Rosbjerg A., Ma Y.J., Skjoedt M.O. (2016). A journey through the lectin pathway of complement-MBL and beyond. Immunol. Rev..

[69] Fernandez-Garcia C.E., Burillo E., Lindholt J.S., Martinez-Lopez D., Pilely K., Mazzeo C., Michel J.B., Egido J., Garred P., Blanco-Colio L.M. (2017). Association of ficolin-3 with abdominal aortic aneurysm presence and progression. J. Thromb. Haemost..

[70] Etienne H., Journe C., Rouchaud A., Senemaud J., Louedec L., Pellenc Q., Coscas R., Gouya L., Dupont S., Michel J.B. (2020). Persistence of Intraluminal Thrombus Makes Saccular Aneurysm More Biologically Active than Fusiform in an Experimental Rat Model. J. Vasc. Res..

[71] Martin-Ventura J.L., Madrigal-Matute J., Martinez-Pinna R., Ramos-Mozo P., Blanco-Colio L.M., Moreno J.A., Tarin C., Burillo E., Fernandez-Garcia C.E., Egido J. (2012). Erythrocytes, leukocytes and platelets as a source of oxidative stress in chronic vascular diseases: Detoxifying mechanisms and potential therapeutic options. Thromb. Haemost..

[72] Sadler J.E. (1998). Biochemistry and genetics of von Willebrand factor. Annu. Rev. Biochem..

[73] Adam D.J., Haggart P.C., Ludlam C.A., von Bradbury A.W. (2003). Willebrand factor and platelet count in ruptured abdominal aortic aneurysm repair. Eur. J. Vasc. Endovasc. Surg..

[74] Hernandez-Ruiz L., Valverde F., Jimenez-Nunez M.D., Ocana E., Saez-Benito A., Rodriguez-Martorell J., Bohorquez J.C., Serrano A., Ruiz F.A. (2007). Organellar proteomics of human platelet dense granules reveals that 14-3-3zeta is a granule protein related to atherosclerosis. J. Proteome Res..

[75] Moxon J.V., Padula M.P., Clancy P., Emeto T.I., Herbert B.R., Norman P.E., Golledge J. (2011). Proteomic analysis of intra-arterial thrombus secretions reveals a negative association of clusterin and thrombospondin-1 with abdominal aortic aneurysm. Atherosclerosis.

[76] Back M., Gasser T.C., Michel J.B., Caligiuri G. (2013). Biomechanical factors in the biology of aortic wall and aortic valve diseases. Cardiovasc. Res..

[77] Tong J., Holzapfel G.A. (2015). Structure, Mechanics, and Histology of Intraluminal Thrombi in Abdominal Aortic Aneurysms. Ann. Biomed. Eng..

[78] Doyle B.J., Callanan A., Burke P.E., Grace P.A., Walsh M.T., Vorp D.A., McGloughlin T.M. (2009). Vessel asymmetry as an additional diagnostic tool in the assessment of abdominal aortic aneurysms. J. Vasc. Surg..

[79] Bluestein D., Niu L., Schoephoerster R.T., Dewanjee M.K. (1996). Steady flow in an aneurysm model: Correlation between fluid dynamics and blood platelet deposition. J. Biomech. Eng..

[80] Basciano C., Kleinstreuer C., Hyun S., Finol E.A. (2011). A relation between near-wall particle-hemodynamics and onset of thrombus formation in abdominal aortic aneurysms. Ann. Biomed. Eng..

[81] Biasetti J., Hussain F., Gasser T.C. (2011). Blood flow and coherent vortices in the normal and aneurysmatic aortas: A fluid dynamical approach to intra-luminal thrombus formation. J. R. Soc. Interface.

[82] Chen C.Y., Anton R., Hung M.Y., Menon P., Finol E.A., Pekkan K. (2014). Effects of intraluminal thrombus on patient-specific abdominal aortic aneurysm hemodynamics via stereoscopic particle image velocity and computational fluid dynamics modeling. J. Biomech. Eng..

[83] Hansen K.B., Arzani A., Shadden S.C. (2015). Mechanical platelet activation potential in abdominal aortic aneurysms. J. Biomech. Eng..

[84] Sarda-Mantel L., Coutard M., Rouzet F., Raguin O., Vrigneaud J.M., Hervatin F., Martet G., Touat Z., Merlet P., Le Guludec D. (2006). 99mTc-annexin-V functional imaging of luminal thrombus activity in abdominal aortic aneurysms. Arterioscler. Thromb. Vasc. Biol..

[85] Rouzet F., Bachelet-Violette L., Alsac J.M., Suzuki M., Meulemans A., Louedec L., Petiet A., Jandrot-Perrus M., Chaubet F., Michel J.B. (2011). Radiolabeled fucoidan as a p-selectin targeting agent for in vivo imaging of platelet-rich thrombus and endothelial activation. J. Nucl. Med..

[86] Bonnard T., Yang G., Petiet A., Ollivier V., Haddad O., Arnaud D., Louedec L., Bachelet-Violette L., Derkaoui S.M., Letourneur D. (2014). Abdominal aortic aneurysms targeted by functionalized polysaccharide microparticles: A new tool for SPECT imaging. Theranostics.

[87] Li B., Juenet M., Aid-Launais R., Maire M., Ollivier V., Letourneur D., Chauvierre C. (2017). Development of Polymer Microcapsules Functionalized with Fucoidan to Target. P-Selectin Overexpressed in Cardiovascular Diseases. Adv. Healthc. Mater..

[88] Chaikof E.L., Dalman R.L., Eskandari M.K., Jackson B.M., Lee W.A., Mansour M.A., Mastracci T.M., Mell M., Murad M.H., Nguyen L.L. (2018). The Society for Vascular Surgery practice guidelines on the care of patients with an abdominal aortic aneurysm. J. Vasc. Surg..

[89] Davies M.J., Murphy W.G., Murie J.A., Elton R.A., Bell K., Gillon J.G., Jenkins A.M., Ruckley C.V. (1993). Preoperative coagulopathy in ruptured abdominal aortic aneurysm predicts poor outcome. Br. J. Surg..

[90] Bradbury A.W., Bachoo P., Milne A.A., Duncan J.L. (1995). Platelet count and the outcome of operation for ruptured abdominal aortic aneurysm. J. Vasc. Surg..

[91] Rajagopalan S., Ford I., Bachoo P., Hillis G.S., Croal B., Greaves M., Brittenden J. (2007). Platelet activation, myocardial ischemic events and postoperative non-response to aspirin in patients undergoing major vascular surgery. J. Thromb. Haemost..

[92] Monaco M., Di Tommaso L., Stassano P., Smimmo R., De Amicis V., Pantaleo A., Pinna G.B., Iannelli G. (2006). Impact of blood coagulation and fibrinolytic system changes on early and mid term clinical outcome in patients undergoing stent endografting surgery. Interact. Cardiovasc. Thorac. Surg..

[93] Englberger L., Savolainen H., Jandus P., Widmer M., Do D., Haeberli A., Baumgartner I., Carrel T.P., Schmidli J. (2006). Activated coagulation during open and endovascular abdominal aortic aneurysm repair. J. Vasc. Surg..

[94] Arnaoutoglou E., Kouvelos G., Papa N., Karamoutsios A., Bouris V., Vartholomatos G., Matsagkas M. (2016). Platelet activation after endovascular repair of abdominal aortic aneurysm. Vascular.

[95] Inoue K., Furuyama T., Kurose S., Yoshino S., Nakayama K., Yamashita S., Morisaki K., Kume M., Matsumoto T., Mori M. (2020). Platelets reflect the fate of type II endoleak after endovascular aneurysm repair. J. Vasc. Surg..

[96] Aoki A., Suezawa T., Sangawa K., Tago M. (2011). Effect of type II endoleaks and antiplatelet therapy on abdominal aortic aneurysm shrinkage after endovascular repair. J. Vasc. Surg..

[97] Johansson P.I., Stensballe J., Rosenberg I., Hilslov T.L., Jorgensen L., Secher N.H. (2007). Proactive administration of platelets and plasma for patients with a ruptured abdominal aortic aneurysm: Evaluating a change in transfusion practice. Transfusion.

[98] Johansson P.I., Swiatek F., Jorgensen L., Jensen L.P., Secher N.H. (2008). Intraoperative platelet and plasma improves survival in patients operated for a rAAA: A follow-up evaluation. Eur. J. Vasc. Endovasc. Surg..

[99] Lunen T.B., Johansson P.I., Jensen L.P., Homburg K.M., Roeder O.C., Lonn L., Secher N.H., Helgstrand U., Carstensen M., Jensen K.B. (2018). Administration of platelets to ruptured abdominal aortic aneurysm patients before open surgery: A prospective, single-blinded, randomised study. Transfus. Med..

[100] Kordzadeh A., Askari A., Parsa A.D., Browne T., Panayiotopoulos Y.P. (2017). The Clinical Implication of Blood Product Transfusion on Morbidity and Mortality of Ruptured Abdominal Aortic Aneurysm. Clin. Appl. Thromb. Hemost..

[101] Keisler B., Carter C. (2015). Abdominal aortic aneurysm. Am. Fam. Phys..

[102] Daugherty A., Cassis L.A. (2004). Mouse models of abdominal aortic aneurysms. Arterioscler. Thromb. Vasc. Biol..

[103] Daugherty A., Manning M.W., Cassis L.A. (2000). Angiotensin II promotes atherosclerotic lesions and aneurysms in apolipoprotein E-deficient mice. J. Clin. Investig..

[104] Pyo R., Lee J.K., Shipley J.M., Curci J.A., Mao D., Ziporin S.J., Ennis T.L., Shapiro S.D., Senior R.M., Thompson R.W. (2000). Targeted gene disruption of matrix metalloproteinase-9 (gelatinase B) suppresses development of experimental abdominal aortic aneurysms. J. Clin. Investig..

[105] Senemaud J., Caligiuri G., Etienne H., Delbosc S., Michel J.B., Coscas R. (2017). Translational Relevance and Recent Advances of Animal Models of Abdominal Aortic Aneurysm. Arterioscler. Thromb. Vasc. Biol..

[106] Lindholt J.S., Vammen S., Fasting H., Henneberg E.W., Heickendorff L. (2000). The plasma level of matrix metalloproteinase 9 may predict the natural history of small abdominal aortic aneurysms. A preliminary study. Eur. J. Vasc. Endovasc. Surg..

